# Arginase Promotes Skeletal Muscle Arteriolar Endothelial Dysfunction in Diabetic Rats

**DOI:** 10.3389/fimmu.2013.00119

**Published:** 2013-05-20

**Authors:** Fruzsina K. Johnson, Robert A. Johnson, Kelly J. Peyton, Ahmad R. Shebib, William Durante

**Affiliations:** ^1^Department of Physiology and Pharmacology, Lincoln Memorial UniversityHarrogate, TN, USA; ^2^Department of Medical Pharmacology and Physiology, University of MissouriColumbia, MO, USA

**Keywords:** arginase, arginine, diabetes, endothelial dysfunction, flow-mediated vasodilation

## Abstract

Endothelial dysfunction is a characteristic feature in diabetes that contributes to the development of vascular disease. Recently, arginase has been implicated in triggering endothelial dysfunction in diabetic patients and animals by competing with endothelial nitric oxide synthase for substrate l-arginine. While most studies have focused on the coronary circulation and large conduit blood vessels, the role of arginase in mediating diabetic endothelial dysfunction in other vascular beds has not been fully investigated. In the present study, we determined whether arginase contributes to endothelial dysfunction in skeletal muscle arterioles of diabetic rats. Diabetes was induced in male Sprague Dawley rats by streptozotocin injection. Four weeks after streptozotocin administration, blood glucose, glycated hemoglobin, and vascular arginase activity were significantly increased. In addition, a significant increase in arginase I and II mRNA expression was detected in gracilis muscle arterioles of diabetic rats compared to age-matched, vehicle control animals. To examine endothelial function, first-order gracilis muscle arterioles were isolated, cannulated in a pressure myograph system, exposed to graded levels of luminal flow, and internal vessel diameter measured. Increases in luminal flow (0–50 μL/min) caused progressive vasodilation in arterioles isolated from control, normoglycemic animals. However, flow-induced vasodilation was absent in arterioles obtained from streptozotocin-treated rats. Acute *in vitro* pretreatment of blood vessels with the arginase inhibitors *N*^ω^-hydroxy-nor-l-arginine or *S*-(2-boronoethyl)-l-cysteine restored flow-induced responses in arterioles from diabetic rats and abolished differences between diabetic and control animals. Similarly, acute *in vitro* pretreatment with l-arginine returned flow-mediated vasodilation in vessels from diabetic animals to that of control rats. In contrast, d-arginine failed to restore flow-induced dilation in arterioles isolated from diabetic animals. Administration of sodium nitroprusside resulted in a similar degree of dilation in arterioles isolated from control or diabetic rats. In conclusion, the present study identifies arginase as an essential mediator of skeletal muscle arteriolar endothelial dysfunction in diabetes. The ability of arginase to induce endothelial dysfunction in skeletal muscle arterioles may further compromise glucose utilization and facilitate the development of hypertension in diabetes.

## Introduction

Diabetes is a progressive metabolic disease that is characterized by an elevation in circulating glucose related to either insulin deficiency (type 1 diabetes) or insulin resistance (type 2 diabetes). Diabetes and its associated complications are a growing concern and represent a serious worldwide public health problem. Vascular disease is the principal cause of morbidity and mortality in patients with diabetes (Kannel and McGee, 1979; Winer and Sowers, 2004). Accelerated atherosclerosis of the large arteries results in increased risk of myocardial infarction, stroke, and limb amputation while microvascular disease is a leading cause of blindness, nephropathy, and neuropathy (Beckman et al., 2002; Porta and Bandello, 2002; Goldberg, 2003; Kikkawa et al., 2003; Duby et al., 2004). Abnormal endothelial function is a salient feature of vascular disease in diabetes that is exemplified by a decrease in nitric oxide (NO) synthesis or bioavailability. In response to shear stress or receptor stimulation, NO is produced by endothelial NO synthase (eNOS) through the oxidation of its substrate, l-arginine. The release of NO by endothelial cells plays a critical role in preserving vascular homeostasis by inhibiting vascular tone, platelet aggregation, leukocyte recruitment and infiltration into the vessel wall, and smooth muscle cell proliferation and migration (see Loscalzo and Welch, 1995; Forstermann and Sessa, 2012). Endothelial dysfunction, including blunted NO-dependent vasodilatory responses, has been documented in patients and animals with diabetes, and is believed to be an important contributor to the pathogenesis of diabetic vascular disease (Durante et al., 1988; Hattori et al., 1991; Tesfamariam and Cohen, 1992; Johnson et al., 1993; Nitenberg et al., 1993).

Although many factors have been implicated in triggering endothelial malfunction, recent studies have identified arginase as a novel mediator of endothelial dysfunction. Arginase is a metalloenzyme that hydrolyzes l-arginine to urea and l-ornithine. There are two distinct isoforms of arginase, arginase I and II, which are encoded by separate genes and share approximately 60% sequence homology (Dizikes et al., 1986; Vockley et al., 1996). Both isoforms of arginase are expressed in the vasculature but their expression is both vessel- and species-dependent (see Durante et al., 2007; Morris, 2009). Arginase elicits endothelial dysfunction by competing with eNOS for substrate l-arginine leading to a deficiency of l-arginine and diminished NO synthesis. Recent work from a number of laboratories has implicated arginase in provoking endothelial dysfunction in several pathological states, including arterial hypertension, pulmonary hypertension, atherosclerosis, aging, and hemorrhagic shock (Xu et al., 2004; Demougeot et al., 2005; Johnson et al., 2005, 2010; Ryoo et al., 2008; Kim et al., 2009). In a seminal study, Romero et al. (2008) demonstrated increased vascular arginase I expression and activity in streptozotocin-treated rats and that arginase contributes to endothelial dysfunction in coronary arteries in this animal model of type 1 diabetes. In addition, it was found that arginase stimulates endothelial dysfunction in myocardial microvessels in type 2 diabetic rats and in coronary arterioles isolated from patients with type 2 diabetes (Beleznai et al., 2011; Gronros et al., 2011). Subsequently, arginase was also shown to impair endothelial function in the aorta, retinal arteries, and corpora cavernosa of streptozotocin-induced type 1 diabetic animals (Toque et al., 2011; El-Bassossy et al., 2012; Romero et al., 2012; Elms et al., 2013). However, the involvement of arginase in mediating endothelial dysfunction in other vascular beds of diabetic animals is not known.

A majority of the studies examining the contribution of arginase to endothelial dysfunction in diabetes utilize acetylcholine to test the vasoactive function of the endothelium. However, a physiological role for acetylcholine in the local regulation of vascular resistance has not been established. Furthermore, in many vascular beds a large portion of acetylcholine-mediated vasodilation is NO-independent (Bolz et al., 1999). Thus, the use of acetylcholine may not fully reveal the nature and physiologic importance of endothelial dysfunction in diabetes. A major *in vivo* stimulus for the synthesis and release of NO by endothelial cells is luminal flow which functions to continuously modulate arterial diameter via changes in shear stress. In order to more fully evaluate the role of arginase in promoting endothelial dysfunction in type 1 diabetes, we determined the expression of arginase I and II in skeletal muscle arterioles in rats treated with streptozotocin or vehicle. In addition, we examined endothelial function in these arterioles in response to a highly relevant physiologic stimulus: luminal flow. Finally, the response of these arterioles to an endothelium-independent vasodilator was also assessed.

## Materials and Methods

### Materials

l-Arginine, d-arginine, glycerol, sodium dodecyl sulfate (SDS), Triton X-100, Tris, sodium acetate, streptozotocin, sodium fluoride, heparin, and sodium nitroprusside were from Sigma-Aldrich (St. Louis, MO, USA). Aprotinin and leupeptin were from Roche Applied Sciences (Indianapolis, IN, USA). *N*^ω^-hydroxy-nor-l-arginine (l-OHNA) and *S*-(2-boronoethyl)-l-cysteine (BEC) were purchased from EMD Biosciences (San Diego, CA, USA). [*Guanido-*^14^C]l-arginine (52 Ci/mmol) was from Amersham Life Sciences (Arlington Heights, IL, USA). All other chemicals were obtained from Fisher Scientific (Houston, TX, USA). Sodium nitroprusside (10 mM) stock solutions were prepared in saline and diluted in modified Krebs buffer immediately before use. l-OHNA (100 μM) and BEC (100 μM) were dissolved in Krebs buffer just before use. The composition of the modified Krebs buffer was (in mM) 118.5 NaCl, 4.7 KCl, 1.4 CaCl_2_, 1.2 KH_2_PO_4_, 1.1 MgSO_4_, 25.0 NaHCO_3_, and 11.1 dextrose.

### Animal model

Adult male Sprague Dawley rats between 12 and 14 weeks of age were purchased from Charles River Laboratories (Wilmington, MA, USA). Diabetes was induced by a single injection of streptozotocin (65 mg/kg, ip) dissolved in sodium citrate (50 mM). Non-diabetic, control animals were injected with an equivalent volume of vehicle. Animals were fed standard rat chow, had free access to drinking water, and were used four weeks after streptozotocin or vehicle administration. All experiments conform to the *Guide for the Care and Use of Laboratory Animals* published by the National Institutes of Health (NIH Publication No. 85–23, revised 1996) and were approved by the institutional care and use committee.

### Hemodynamic and metabolic measurements and tissue extractions

Four weeks after the streptozotocin or vehicle administration, rats were weighed and injected intraperitoneally with ketamine (100 mg/kg) and zylazine (7.5 mg/kg) (Butler Schein Animal Health Corporation, Dublin, OH, USA), and a carotid arterial catheter implanted for blood sample collection and blood pressure measurement. Blood samples were drawn for immediate determination of blood glucose (Accu-Chek Compact, Roche Diagnostics, Indianapolis, IN, USA), glycated hemoglobin (HbA1c, DCA 2000+ Analyzer, Bayer, Pittsburgh, PA, USA), and cholesterol (CardioChek PA Analyzer, Polymer Technology Systems, Inc., Indianapolis, IN, USA). Blood pressure was measured using a pressure transducer (TSD 104A, Biopac Systems, Santa Barbara, CA, USA) coupled to a polygraph system (Biopac Systems, Santa Barbara, CA, USA) and a personal computer. Animals were then heparinized (1000 U/kg, iv) and the thoracic aorta and gracilis anticus muscles removed and placed into ice-cold modified Krebs buffer or frozen in liquid nitrogen and stored at −70°C for later use.

### Arginase activity

Arginase activity was determined by monitoring the formation of [^14^C]urea from [*guanido-*^14^C]l-arginine, as we previously reported (Peyton et al., 2009). Blood vessels were sonicated in Tris buffer (10 mm, pH 7.4) containing Triton X-100 (0.4%), leupeptin (10 mg/mL), and aprotinin (10 mg/mL). Lysates (100 μg) were added to an equal volume of Tris buffer (10 mM, pH 7.4) containing MnCl_2_ (10 mM) and arginase was activated by heating for 10 min at 56°C. The arginase reaction was initiated by adding Tris buffer containing l-arginine (10 mM) and [*guanido-*^14^C]l-arginine (0.25 Ci), and samples were incubated at 37°C for 30 min. Reactions were terminated by adding ice-cold sodium acetate buffer (250 mM, pH 4.5) containing urea (100 mM). [^14^C]Urea was separated from basic amino acids by Dowex chromatography and [^14^C]urea formation determined by scintillation counting.

### Arginase expression

Arginase expression was determined by quantitative real-time PCR. Total RNA was isolated from gracilis muscle arterioles using TRIzol reagent and quantified by absorbance spectroscopy. cDNA was synthesized with 1 μg of RNA using iScript cDNA synthesis kits (Bio-Rad, Hercules, CA, USA). Quantitative real-time PCR was carried out using a SYBR Green Supermix (Bio-Rad, Hercules, CA, USA) and arginase primers using the Bio-Rad CFX96 system (Sasatomi et al., 2008). Thermal cycling was performed at 95°C for 10 min followed by 40 cycles at 95°C for 15 s and 60°C for 1 min. Reactions were performed in triplicate. Relative expression of arginase was analyzed using the delta–delta Ct method and results normalized with respect to 18S rRNA.

### Isolated microvessel experiments

Segments of first-order gracilis muscle arterioles were isolated by microdissection and cannulated at both ends with glass micropipettes in a vessel chamber (Living Systems Instrumentation, Burlington, VT, USA). The vessel chamber was continuously perfused with Krebs buffer equilibrated with a gas mixture of 14% O_2_ and 5% CO_2_, balanced with N_2_, in a non-recirculating system. For internal diameter measurements, the vessel chamber was mounted on a stage of an inverted microscope (TS 100-F, Nikon Instruments, Melville, NY, USA) fitted with a CCD video camera. The camera was connected to a personal computer equipped with video dimensioning software (ImagePro Express, Media Cybernetics, Bethesda, MD, USA). Images were collected at 1 frame/s and stored as digital files for documentation.

To study agonist-induced dilation, the proximal micropipette was connected to a pressure servo controller (Living Systems Instrumentation, Burlington, VT, USA) and the distal micropipette was connected to a closed stopcock to achieve 80 mmHg constant luminal pressure with no flow. Following a 60 min stabilization period, peak responses of arterioles to cumulative additions of sodium nitroprusside (10–1000 nM) were determined by adding the drug to the superfusion buffer. To study flow-induced dilation, both the proximal and distal micropipettes were connected to pressure servo controllers and to an inline micro flowmeter (Living Systems Instrumentation, Burlington, VT, USA). During a 60 min stabilization period, both proximal and distal pressures were adjusted to 80 mmHg with no luminal flow. In order to establish graded levels of luminal flow (0–50 μL), proximal and distal pressures were adjusted equally in the opposite direction maintaining midline pressure at 80 mmHg. In some experiments, arginase inhibitors or arginine was added to the superfusion buffer 20 min before arterioles were exposed to luminal flow. In order to limit the possible loss of vascular l-arginine from vessels perfused with buffer devoid of l-arginine that may potentially mask differences between the two groups of animals, the duration of experiments was restricted to a single flow-response curve per vessel.

### Statistics

Results are expressed as mean ± SEM. Statistical analyses were performed with the use of a Student’s two-tailed *t*-test and an analysis of variance with the Tukey *post hoc* test when more than two treatment regimens were compared. *p*-Values <0.05 were considered statistically significant.

## Results

**Figure 1:**
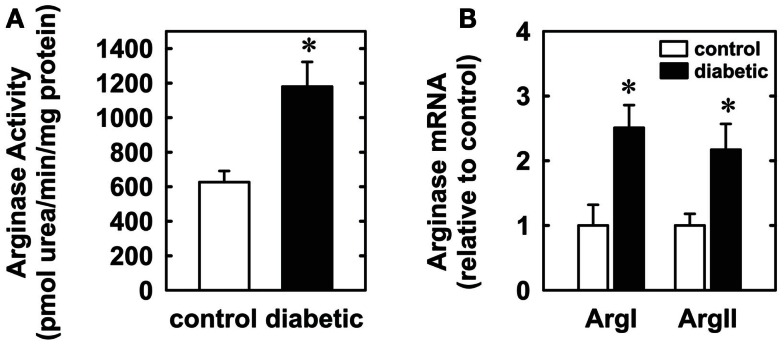
**Diabetes stimulates vascular arginase activity and expression in rats**. Diabetes increases arginase activity in the aorta **(A)** and arginase I and II mRNA expression in gracilis muscle arterioles **(B)**. Results are expressed as mean ± SEM (*n* = 4–5). *Statistically significant effect of diabetes (*p* < 0.05).

Four weeks after administration of streptozotocin, rats develop overt diabetes as reflected by elevated fasting blood glucose and glycated hemoglobin levels compared to vehicle-treated control animals (Table 1). In addition, body weights of streptozotocin-diabetic rats were significantly lower than control animals. However, there was no significant difference in blood pressure, heart rate, or circulating cholesterol levels between control and diabetic rats. Vascular arginase activity was markedly increased in diabetic rats by nearly twofold (Figure 1A). The rise in aortic arginase activity in diabetic animals was associated with a significant increase in the expression of both arginase I and II mRNA in gracilis muscle arterioles (Figure 1B).

**Table 1:** **Metabolic and hemodynamic parameters of control and diabetic rats**.

	Control	Diabetic
Body weight (g)	472 ± 10	288 ± 14*
Blood pressure (mmHg)	122 ± 9	114 ± 12
Heart rate (beats/min)	328 ± 22	302 ± 15
Glucose (mg/dL)	98 ± 8	411 ± 32*
Glycated hemoglobin (%)	3.9 ± 0.1	7.9 ± 0.2*
Cholesterol (mg/dL)	78 ± 6	87 ± 11

*Results are means ± SEM (*n* = 14–17)*.

*Statistically significant effect of diabetes (*p* < 0.01).

**Figure 2:**
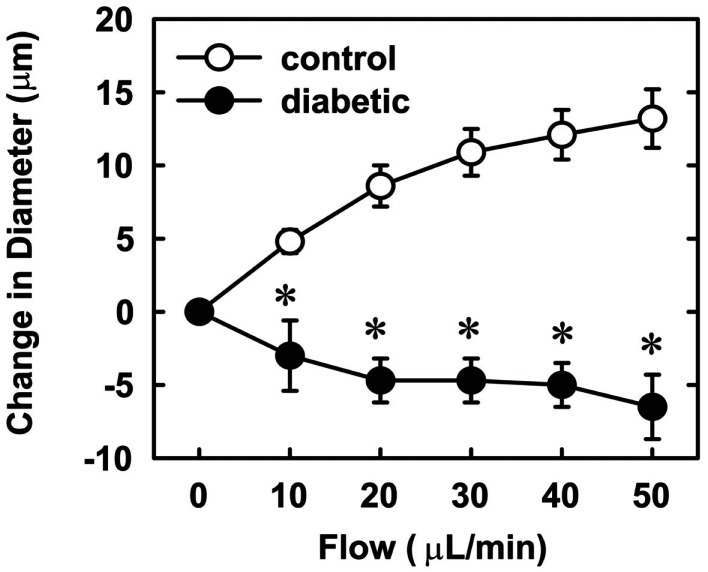
**Diabetes abolishes flow-dependent increases in internal diameter of gracilis muscle arterioles**. Arterioles were isolated from control (open circles) or diabetic (closed circles) rats and exposed to graded levels of luminal flow. Results are expressed as mean ± SEM (*n* = 3–5). *Statistically significant effect of diabetes (*p* < 0.01).

**Figure 3:**
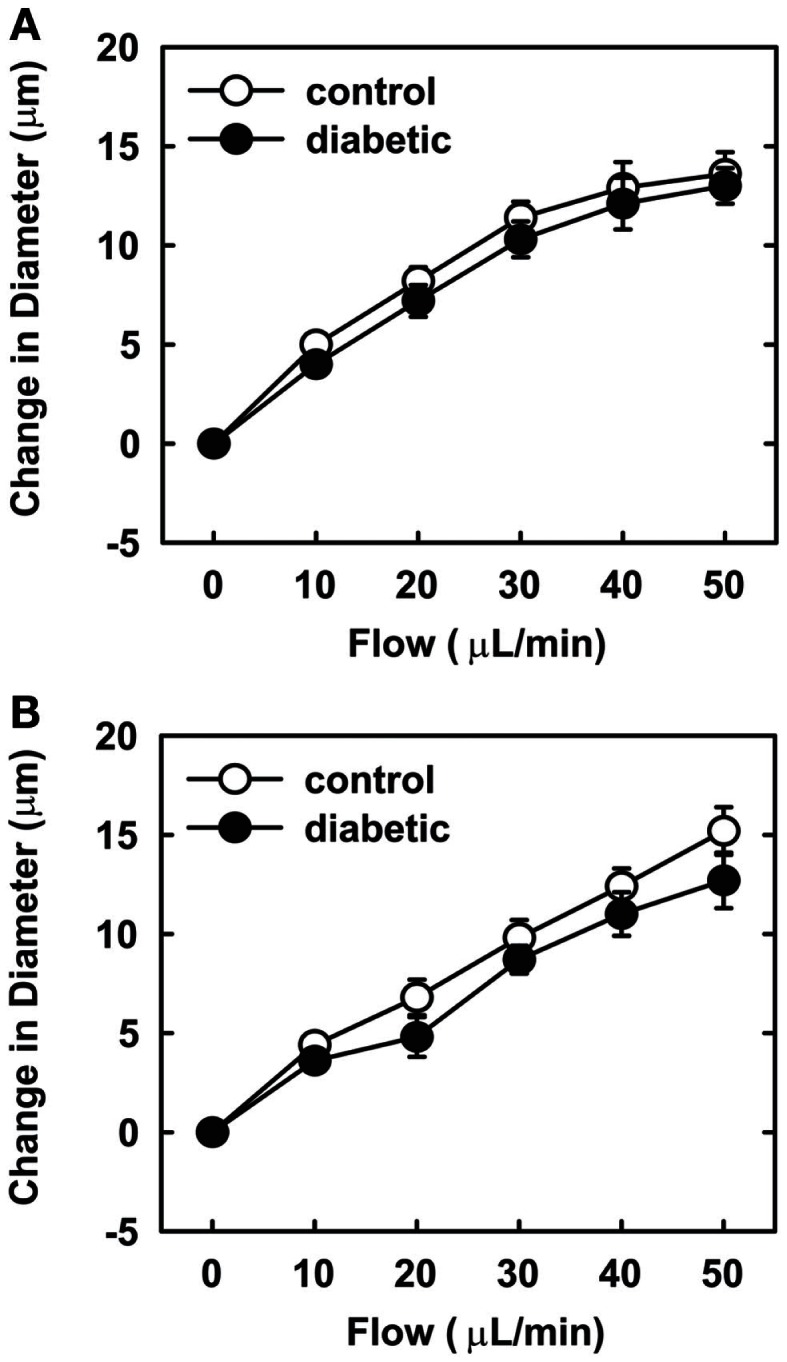
**Arginase inhibition restores flow-dependent increases in internal diameter of gracilis muscle arterioles in diabetes**. Arterioles isolated from control (open circles) or diabetic (closed circles) rats were treated *in vitro* with the arginase inhibitors nor-NOHA (100 μM) **(A)** or BEC (100 μM) **(B)** and then exposed to graded levels of luminal flow. Results are expressed as mean ± SEM (*n* = 5).

**Figure 4:**
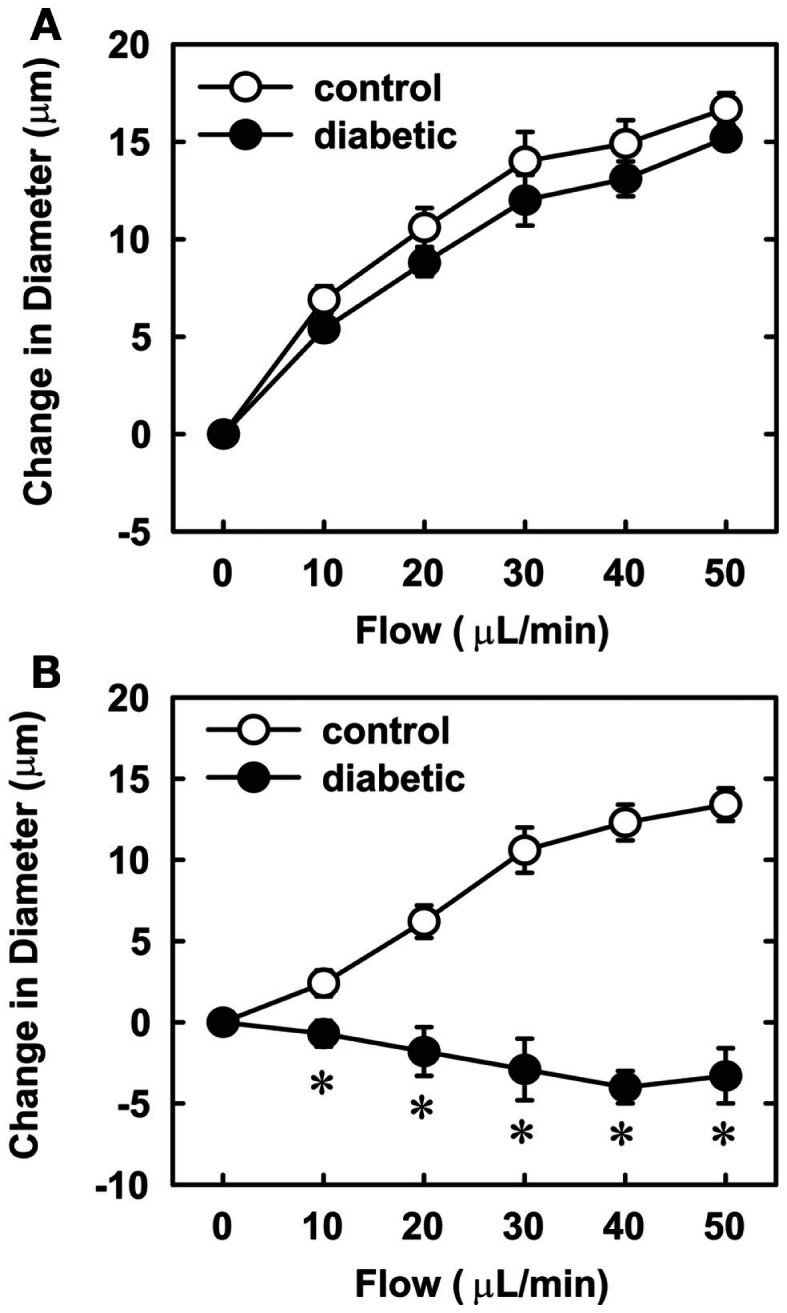
**Effect of arginine on flow-dependent increases in internal diameter of gracilis muscle arterioles**. Arterioles isolated from control (open symbol) or diabetic (closed symbol) rats were treated *in vitro* with the l-arginine (1 mM) **(A)** or d-arginine (1 mM) **(B)** and then exposed to graded levels of luminal flow. Results are expressed as mean ± SEM (*n* = 4–5). *Statistically significant effect of diabetes (*p* < 0.01).

**Figure 5:**
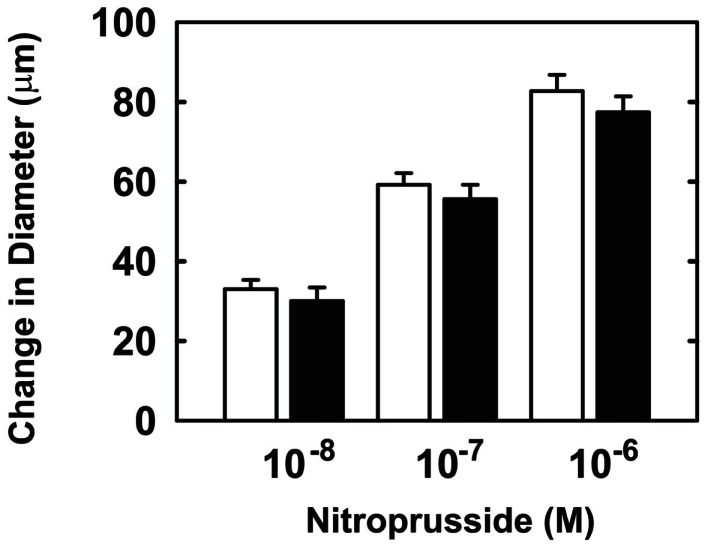
**Sodium nitroprusside-mediated increases in internal diameter of gracilis muscle arterioles**. Arterioles isolated from control (open symbol) or diabetic (closed symbol) rats were exposed to various concentrations of sodium nitroprusside. Results are expressed as mean ± SEM (*n* = 5).

Endothelial function was examined in isolated skeletal muscle arterioles. Increases in luminal flow (0–50 μL/min) resulted in progressive vasodilation in arterioles isolated from control rats (Figure 2). However, flow-mediated vasodilation was absent in arterioles isolated from diabetic animals. In fact, a slight vasoconstrictor effect was noted in diabetic vessels subjected to luminal flow. Since arginase expression was elevated in gracilis muscle arterioles of diabetic rats, we examined if arginase was responsible for impairing endothelial function in these animals. Acute *in vitro* pretreatment of blood vessels with the arginase inhibitors, l-OHNA (100 μM) or BEC (100 μM), restored flow-induced responses in arterioles from diabetic animals and abolished differences between the two groups of animals (Figures 3A,B). Similarly, acute *in vitro* pretreatment of vessels with the arginase and NO synthase substrate, l-arginine (1 mM), reinstated flow-induced dilation in arterioles obtained from diabetic rats and abrogated the difference between control and diabetic animals (Figure 4A). In contrast, d-arginine (1 mM), which is not a substrate for either enzyme, failed to restore flow-mediated responses in arterioles from diabetic rats (Figure 4B). Finally, the responsiveness of arteriole smooth muscle to NO was tested by treating vessels with the NO donor, sodium nitroprusside. Sodium nitroprusside dilated arterioles isolated from control or diabetic animals in a similar concentration-dependent manner (Figure 5).

## Discussion

In the present study, we found that vascular arginase activity is elevated in streptozotocin-treated diabetic rats and this is associated with a significant increase in the expression of arginase I and II in gracilis muscle arterioles. In addition, we discovered that luminal flow-induced vasodilation is severely compromised in gracilis muscle arterioles isolated from diabetic rats while the vasodilator response to the endothelium-independent agonist, sodium nitroprusside, is preserved in these vessels. We also found that acute *in vitro* pretreatment with arginase inhibitors restores flow-induced vasodilation and abolishes the difference between control and diabetic arterioles. Similarly, acute *in vitro* pretreatment with the arginase and eNOS substrate, l-arginine, but not the inactive d isomer, restores flow-mediated vasodilation and eliminates the difference between the two groups of animals. These findings suggest that increased arginase activity contributes to skeletal muscle arteriolar endothelial dysfunction in type 1 diabetes by restricting the availability of l-arginine.

Diabetes was induced in our study by a single injection of streptozotocin. This glucose moiety selectively destroys the insulin-producing β-cells of the pancreas leading to rapid insulin-deficiency and diabetes. This animal model displays polydipsia, polyuria, hyperglycemia, and weight loss that are similar to the clinical symptoms found in diabetic patients (Wei et al., 2003; Akbarzadeh et al., 2007). In agreement with this, we found that rats treated with streptozotocin develop frank diabetes 4 weeks after treatment as indicated by raised fasting blood glucose and glycated hemoglobin concentrations and loss in body weight. The significant decline in body weight is more pronounced in adult animals in this model and likely reflects the loss of glucose in the urine and an inability to metabolize carbohydrates and a shift to fat metabolism leading to depletion of fat stores (Hoybergs et al., 2008).

Our finding that vascular arginase activity is increased in type 1 diabetic rats is consistent with recent studies in streptozotocin-treated rats and mice where elevated arginase activity was reported in the liver, aorta, kidney, macrophages, retina, and corpora cavernosa (Romero et al., 2008, 2012; Morris et al., 2011; Toque et al., 2011; Sun et al., 2012; Elms et al., 2013). For the first time, we also show that diabetes induces the expression of both arginase I and II in gracilis muscle arterioles. This novel observation contrasts with earlier studies showing that diabetes selectively stimulates arginase I or II expression in different tissues, further underscoring the tissue and vessel-dependent pattern of arginase expression (see Durante et al., 2006; Morris, 2009). Interestingly, we previously demonstrated that arginase I and II are also both elevated in rat gracilis muscle arterioles following the development of salt-sensitive hypertension, suggesting that arginase I and II are regulated in a coordinate fashion in these vessels (Johnson et al., 2005). The induction of arginase in diabetes may occur via multiple mechanisms. Since insulin was recently demonstrated to suppress plasma arginase activity in diabetic patients, the reduction or lack of circulating insulin may contribute to the increase in arginase activity in type 1 diabetes (Kashyap et al., 2008). However, hyperglycemia also appears to be involved in the widespread induction of arginase observed in diabetes as exposure of vascular cells to high concentrations of glucose triggers a rise in arginase activity (Durante et al., 2006; Romero et al., 2008). The ability of hyperglycemia to augment arginase activity likely occurs via the RhoA pathway, which can stimulate the induction or activation of both arginase isoforms (Ming et al., 2004; Romero et al., 2008).

We also found that endothelial function is markedly impaired in skeletal muscle arterioles from diabetic rats. Endothelial function was examined using a physiologically relevant stimulus, fluid flow, in first-order gracilis muscle arterioles. We previously reported that flow-mediated vasodilation in gracilis muscle arterioles from normoglycemic rats is completely abolished by eNOS inhibition, indicating that NO is responsible for flow-induced vasodilation in these vessels (Johnson and Johnson, 2008). While arterioles from control rats exhibit pronounced vasodilation in response to luminal flow, flow-induced vasodilation is absent in arterioles isolated from diabetic animals, suggesting that endothelial NO synthesis or bioavailability is dramatically reduced in these vessels. Arterioles from diabetic rats respond as well as arterioles from control animals to the NO donor, sodium nitroprusside, indicating that the sensitivity of arteriolar smooth muscle to NO is unchanged in diabetic animals. Our finding that gracilis muscle arterioles from streptozotocin-treated diabetic rats display a modest vasoconstrictor response to flow has also been reported in mesenteric arteries from these animals and may be linked to diminished NO production (Tribe et al., 1998). To test whether arginase contributes to impaired endothelial function in type 1 diabetes, we employed two distinct arginase inhibitors, l-OHNA and BEC, which are highly potent inhibitors of arginase I and II (Christianson, 2005). We found that acute pretreatment with either l-OHNA or BEC restores flow-induced dilation in arterioles from diabetic rats to levels seen in vessels from control animals. Taken in conjunction with our findings that vascular arginase I and II expression are increased in diabetic arterioles, these results suggest that arginase contributes to gracilis muscle arteriolar NO dysfunction in type 1 diabetes. Our current experimental findings build on previous work in the vasculature of the heart, retina, and corpora cavernosa (Romero et al., 2008, 2012; Toque et al., 2011) and are also supported by a recent clinical study showing arginase inhibition markedly improves endothelium-dependent vasodilation in the forearm of patients with type 2 diabetes and coronary artery disease (Shemyakin et al., 2012). Thus, arginase-mediated endothelial dysfunction may be a characteristic feature of diabetes that encompasses many vascular beds.

Our finding that arginase inhibition restores endothelium-dependent vasodilation of skeletal muscle arterioles from diabetic animals suggests that arginase may modulate l-arginine availability for NO synthesis in diabetes. Consideration of the enzyme kinetics for arginase and eNOS, indicates that arginase can effectively compete with eNOS for substrate l-arginine (Wu and Morris, 1998). Consistent with a role for arginase in depleting substrate for eNOS, vascular l-arginine concentrations are significantly reduced in streptozotocin-treated diabetic rats (Pieper and Dondlinger, 1997). Moreover, we showed that acute administration of l-arginine mimics the effect of arginase inhibition. l-Arginine restores flow-induced vasodilation in arterioles from diabetic rats whereas the inactive isomer, d-arginine, has no effect. The induction of vascular arginase and the subsequent depletion of l-arginine may also explain the ability of exogenously administered l-arginine to restore endothelial function and NO synthesis in both chemical and genetic animal models of type 1 diabetes (Pieper and Dondlinger, 1997; Pieper et al., 1997; Kohli et al., 2004). Furthermore, arginase-mediated decreases in intracellular l-arginine may negatively impact endothelial function by sensitizing endothelial cells to the endogenous eNOS inhibitor, asymmetric dimethylarginine, which is elevated in streptozotocin-treated rats (Lin et al., 2002). Finally, arginase-mediated depletion of arginine may further compromise endothelial function in diabetes by uncoupling eNOS (Kim et al., 2009).

The ability of arginase to inhibit endothelial function in skeletal muscle arterioles in diabetes is of pathological significance. Skeletal muscle arterioles contribute greatly to peripheral resistance and consequently are major determinants of blood pressure. Thus, arginase-mediated impairment of skeletal muscle arteriolar function may contribute to the development of hypertension in diabetes. Although blood pressure not increased four weeks after streptozotocin administration in our study, a longer duration of diabetes is associated with the development of hypertension in this animal model. Recently, El-Bassossy et al. (2012) reported that diabetic rats develop a significant increase in systolic and diastolic blood pressure 8 weeks following streptozotocin treatment. Notably, administration of arginase inhibitors for the last 6 weeks significantly reduced the developed elevation in diastolic blood pressure in these animals, illustrating a role for arginase in diabetes-associated hypertension. Since skeletal muscle represents a major site of insulin-dependent glucose uptake and utilization, arginase-mediated endothelial dysfunction may limit skeletal muscle perfusion and exacerbate hyperglycemia in diabetes. In support of this proposal, arginase inhibition reduces the rise in serum glucose and advanced glycation end products in type 1 diabetic rats (El-Bassossy et al., 2012), while arginase II deletion improves glucose tolerance and insulin sensitivity in type 2 diabetic mice (Ming et al., 2012). Thus, arginase-mediated endothelial dysfunction of skeletal muscle arterioles may promote the vascular and metabolic derangements observed in diabetes.

In conclusion, the present study identifies arginase as a critical mediator of skeletal muscle arteriolar endothelial dysfunction in diabetes. Flow-induced vasodilation is abolished in gracilis muscle arterioles of streptozotocin-treated rats and this is associated with an increase in arteriolar arginase I and II expression. Moreover, pretreatment of blood vessels with arginase inhibitors or l-arginine fully restores flow-induced vasodilation in arterioles from diabetic rats. These results provide novel insight into the mechanism by which skeletal muscle arteriolar function is compromised in diabetes, and establishes arginase as a potential therapeutic target in treating vascular and metabolic disorders in diabetes.

## Conflict of Interest Statement

The authors declare that the research was conducted in the absence of any commercial or financial relationships that could be construed as a potential conflict of interest.
